# Remarks on the Religious Persuasion, Degree of Education, and Employments of Persons Affected with Insanity

**Published:** 1855-10-01

**Authors:** John Webster

**Affiliations:** Fellow of the Royal College of Physicians, &c.


### REMARKS ON THE RELIGIOUS PERSUASION, DEGREE OF EDUCATION, AND EMPLOYMENTS OF PERSONS AFFECTED WITH INSANITY.

BY JOHN WEBSTER, M.D., F.R.S.,

*Fellow of the Royal College of Physicians, &c.*

TWELVE years ago, the managing committee of Bethlem Hospital adopted, at my recommendation, a series of tables illustrating various important facts respecting the lunatics admitted and placed under treatment in that institution. Since the period mentioned, these valuable and instructive records have been regularly continued, and now constitute highly useful documents for reference. During the period embraced by the elaborate statistical returns thus accumulated, 3599 insane patients have been admitted into the curable wards of the above named establishment, all being only recently attacked with mental disease. Of these, the largest proportion were females, the exact number of that sex being 2074; whereas only 1325 were male lunatics, the ratio thus amounting to fifty-six per cent. more of the former than the latter division; which statement conclusively demonstrates the greater prevalence of insanity among women than men, throughout the general metropolitan population.

Much interesting information may be derived from a careful examination of the above tables; and if the aggregate facts are properly arranged, some curious practical deductions may be thereby likewise obtained. Believing a few cursory remarks in reference to the religious persuasion, degree of education, and different employments of the numerous lunatic persons of both sexes received at Bethlem Hospital, during the last twelve years, will not be considered unimportant, or out of place in the *Psychological Journal*, I would therefore beg leave to direct its readers' attention to the subject now proposed for discussion. Of course, it is impossible to deduce, from present data, any special inferences in regard to the comparative frequency of lunacy, amongst different religious persuasions: its prevalence in ignorant contrasted with persons of education: or the influence which particular kinds of employments may actually exert upon individuals. Some general approximation to the truth can only be expected. Nevertheless, the large array of figures which have been thus brought into juxtaposition, cannot prove otherwise than extremely useful to every investigator.

Influenced by such motives, I will therefore proceed to examine the part first proposed for inquiry; namely, the religious persuasion of lunatics admitted. As might be expected, the largest number belonged to the Church of England, nearly two-thirds, or seventy-three per cent. of the whole admissions being members of that religious community. More females than males were however enumerated in this category, or 972 of the latter sex to 1559 of the former, and so making a total of 2531 individuals belonging to the national religious establishment. The next most numerous body were Independents, of whom 234 came under treatment, 81 being males, and nearly twice as many, or 153 females. Wesleyans follow: the total of that persuasion being 144 persons, comprising 57 of the male, and 87 of the other sex. Baptists appear next in the scale: these comprise 129 persons, or 50 males and 79 females. Afterwards Roman Catholics, of whom the total admissions reached 120, giving 50 male and 70 female lunatics. Then Presbyterians: 73 members of that body having been received, the sexes being nearly equal, or 34 males to 39 females. The Jews occupy the next position: 47 Israelites, of whom the sexes were almost the same in number, or 23 male to 24 female lunatic patients being included in the division. Dissenters supplied only 17 individuals, 10 being male, and only seven female patients; whilst the remainder, although comprising varied and even very opposite shades of religious opinions, were, however, few in number taken specifically. Thus, six were Unitarian; five being men, and only one woman. Plymouth Brethren also supplied six patients, of whom two were males and four females. Again, Lady Huntingdon likewise furnished six proselytes, two being male, and four female lunatics. Deists and Atheists actually amounted to five persons, but all men. Swedenborgians gave two males. Ranters, one patient of each sex. Quakers occupied a similar position. Moravians supplied one male. Sandemanians, Socialists, and the Greek Church also one male patient each. And lastly, Antinomians, Brownites, and Irvingites, were respectively represented by one insane female belonging to each of these so-called religious communities. The remaining 27 male and 43 female lunatic inmates treated at Bethlem Hospital, making a total of 70 persons, being reported as "not ascertained" in respect of their specific denomination.

The mental culture, or degree of education, which the large number of persons comprised in the present inquiry had acquired, previous to becoming insane, and so rendering them proper subjects for confinement, now comes under review; and upon this interesting question some curious information has been acquired. Of the total 3599 inmates received into this institution, 208 were lunatics who had previously obtained a superior education, and some even were exceedingly accomplished; the sexes being nearly equal, or 102 males to 106 females. With reference to sex, the proportion of highly educated persons was greater amongst men than women, seeing the ratio was one in every thirteen of the former, but only one in every twenty of the latter division of inmates. Patients whose education was reported good, that is, could both read and write, amounted altogether to 890 individuals, or one-fourth of the entire number admitted. The figures representing each sex being almost similar, or 425 males to 465 females, which however gives respectively one in every three male lunatics admitted able to read and write, whilst only two in every nine women had even acquired that limited education.

Those who could only read, but were wholly incapable of writing, both sexes included, amounted to the very large number of 1887 persons, making not less than 52·40 per cent., or upwards of half the entire admissions. Here, again, the females appeared even more ignorant than the male patients, 1181 of the former being able only to read, whilst 706 males came within that category. Lastly, 417 human beings, out of 3599 once endued with reason, but now deprived of that inestimable blessing, had never received any kind of mental education whatever, being steeped, ever since birth, in the most crass ignorance, and wholly incapable either of wielding a pen, or still less knowing the alphabet.

So lamentable a circumstance, that nearly one-eighth of the aggregate patients admitted into this charity, where parish paupers are seldom received, and the recipients of its bounty are, in a large proportion, connected with tradesmen or the mechanical classes of society, should be found in such an abject condition as neither to know how to write nor even to read, speaks trumpet-tongued against the negligence of parents, guardians, and parochial authorities; whilst it clearly shows much remains to be accomplished ere popular ignorance shall be entirely dispelled. It is also remarkable that the number of females who were thus classified, or living hitherto, so to speak, in perfect Cimmerian darkness, was much greater than amongst males, the amount being 325 females, or 15·66 per cent. of the entire admissions; whereas, only 92 male lunatics were similarly situated, being nearly seven per cent, or under half the ratio recorded amongst the other sex. Such facts are exceedingly interesting; but whether insanity is more liable to affect ignorant minds, and those never improved by any mental culture, cannot be authoritatively asserted from the data thus hitherto obtained, although the present statements tend, in some measure, to prove such an inference; seeing so many persons, especially females, whose intellectual faculties had never been cultivated, nor attempts made to enable them to acquire knowledge in the usual manner, come within this category. It seems, however, a generally received and well-founded opinion, that persons of education, or individuals whose intellects have been properly trained, especially in the exact sciences, and embued with practical information, but not weakened through inane absurd accomplishments, however fashionable, or their feelings excited by meretricious systems of teaching, are far likelier to continue mentally sane, than parties belonging to the uncultivated and ignorant classes. Psychologists may therefore fairly infer, even from the facts now reported, that ignorance promotes insanity; and the human mind, which has not been subjected to any kind of discipline, is more apt to be afflicted by mental disease, than, comparatively speaking, where parties are differently constituted.

The remaining point to which I would now specially direct attention, is the kind of occupation, or previous employment, of the different insane patients placed under treatment at Bethlem Hospital. The list is both large and curious; whilst it demonstrates, although several handicrafts give few victims, nevertheless, amongst various occupations contained in this table, the sufferers from mental alienation were more numerous in some employments than in others, respecting which feature I shall afterwards append one or two general observations, chiefly based upon facts the subsequent document contains.

*No. 1.—Occupations of Male Patients.*

| | | | | | |
|---|---|---|---|---|---|
| Accountants | 2 | Authors | 3 | Blind Maker | 1 |
| Actor | 1 | Bakers | 33 | Bookbinders | 9 |
| Agents | 6 | Barristers | 2 | Booksellers | 5 |
| Architects | 7 | Basket Makers | 2 | Brassfounders | 3 |
| Army Clothier | 1 | Billiard Marker | 1 | Brokers | 12 |
| Artists | 8 | Boatbuilders | 4 | Brewers | 3 |
| Attorneys | 4 | Boiler Maker | 1 | Bricklayers | 13 |
| Auctioneer | 1 | Blacksmiths | 14 | Brush Makers | 2 |

| | | | | | |
|---|---|---|---|---|---|
| Builders | 4 | Gasfitter | 1 | Porters | 25 |
| Cane Maker | 1 | Glass Cutter | 1 | Postmen | 3 |
| Carpenters | 81 | Glover | 1 | Printers | 18 |
| Carriers | 3 | Goldsmiths | 10 | Publicans | 25 |
| Carvers and Gilders | 6 | Greengrocers | 7 | Railway Servants | 4 |
| Cabmen | 6 | Grocers | 28 | Relieving Officer | 1 |
| Carmen | 3 | Hair Dressers | 12 | Rope Maker | 1 |
| Cellarmen | 2 | Hatters | 8 | Saddlers | 5 |
| Chair Makers | 4 | Hawkers | 16 | Sailcloth Maker | 1 |
| Chemists | 8 | Hosiers | 2 | Sailors | 29 |
| Cheesemongers | 6 | House Decorators | 2 | Salesman | 1 |
| Chinamen | 2 | Ironfounder | 1 | Sawyers | 11 |
| Clerks | 83 | Ironmongers | 3 | Schoolmasters | 27 |
| Clergymen | 9 | Japanners | 2 | Servants | 32 |
| Clothier | 1 | Labourers | 80 | Shirt Cutters | 2 |
| Coach Makers | 9 | Land Surveyor | 1 | Shoemakers | 48 |
| Coachmen | 6 | Last Maker | 1 | Shepherds | 3 |
| Coal Dealers | 5 | Lath Renders | 4 | Shopmen | 10 |
| Collar Maker | 1 | Law Stationers | 2 | Silk Merchants | 2 |
| Commercial Travellers | 7 | Law Writers | 6 | Slater | 1 |
| Confectioners | 2 | Leather Dressers | 4 | Soldiers | 2 |
| Contractor | 1 | Lighthouse Keeper | 1 | Spring Maker | 1 |
| Cooks | 2 | Lightermen | 4 | Sugar Refiner | 1 |
| Coopers | 6 | Livery Stablers | 5 | Surveyors | 5 |
| Copperplate Printer | 1 | Mast Maker | 1 | Stationers | 2 |
| Cork Cutter | 1 | Medical | 22 | Stokers | 2 |
| Corn Chandlers | 4 | Merchants | 2 | Stonemasons | 2 |
| Corn Dealer | 1 | Miners | 2 | Storekeeper | 1 |
| Curriers | 11 | Millers | 3 | Students | 4 |
| Cutler | 1 | Millwrights | 2 | Tailors | 35 |
| Dairymen | 3 | Musicians | 17 | Tablecloth Maker | 1 |
| Dealer in Hides | 1 | Newsvender | 1 | Tanner | 1 |
| Dentist | 1 | Oilmen | 2 | Tidewaiters | 2 |
| Drapers | 4 | Officers of Army | 7 | Tinmen | 3 |
| Draughtsman | 2 | Officers of Customs, &c. | 3 | Tobacconists | 12 |
| Drovers | 2 | Officer of Navy | 1 | Truss Maker | 1 |
| Dyers | 2 | Old Clothes Dealers | 7 | Upholsterers | 3 |
| Egg Merchant | 1 | Omnibus Proprietor | 1 | Watchmakers | 11 |
| Engineers | 8 | Opticians | 5 | Waiters | 6 |
| Engravers | 11 | Painters & Plumbers | 25 | Weavers | 11 |
| Farmers | 34 | Parish Clerks | 2 | Wheelwrights | 2 |
| Fishermen | 3 | Pattern Designers | 4 | Whitesmiths | 2 |
| Fishmongers | 16 | Pianoforte Makers | 8 | Wine Merchants | 2 |
| Flower Makers | 3 | Pilot | 1 | Wood Cutters | 2 |
| French Polishers | 3 | Pipe Maker | 1 | No profession | 21 |
| Fruiterer | 1 | Plasterers | 3 | Not ascertained | 84 |
| Furriers | 4 | Pocket-book Maker | 1 | | |
| Gamekeeper | 1 | Policemen | 14 | Total | 1325 |
| Gardeners | 16 | | | | |

*No. 2.—Occupations of Female Patients.*

| | | | | | |
|---|---|---|---|---|---|
| Annuitants | 2 | Confectioner | 1 | Fur Dresser | 1 |
| Artists | 3 | Cooks | 5 | Glovers | 2 |
| Baker | 1 | Corn Dealers | 3 | Governesses | 62 |
| Barmaids | 5 | Dairywomen | 6 | Greengrocer | 1 |
| Book Folders | 2 | Dressmakers | 148 | Hat Liners | 2 |
| Bonnet Makers | 12 | Eating-house Keeper | 1 | House Agent | 1 |
| Button Coverer | 1 | Embroiderers | 3 | Housekeepers | 10 |
| Carrier | 1 | Envelope Maker | 1 | Hawkers | 2 |
| Charwomen | 7 | Flower Makers | 3 | Keeper of an Office | 1 |
| Clothworker | 1 | Furniture Brokeress | 1 | Lace Makers | 7 |

| | | | | | |
|---|---|---|---|---|---|
| Lady's Companions... | 2 | Shopwomen ... ... | 8 | Wives, Widows, and Daughters of Professional Men, Officers, and Merchants | 47 |
| Lady's Maids ... ... | 14 | Silk Winders ... ... | 3 | Wives, Widows, and Daughters of Clerks and Tradesmen .. | 582 |
| Laundresses ... ... | 31 | Singer ... ... ... | 1 | Wives, Widows, and Daughters of Labourers, Servants, and Mechanics ... | 465 |
| Linendraper ... ... | 1 | Stay Makers ... ... | 3 | No occupation, or Not ascertained ... | 164 |
| Lodging-house-keepers | 25 | Stock Maker ... ... | 1 | Total ... | ...2074 |
| Map Colourer ... ... | 1 | Straw Plaiters ... ... | 2 | | |
| Midwife ... ... ... | 1 | Tambour Worker ... | 1 | | |
| Needlewomen ... ... | 64 | Toll-gate Keeper ... | 1 | | |
| Nurses ... ... ... | 26 | Upholstresses ... ... | 2 | | |
| Organist ... ... ... | 1 | Umbrella Maker ... | 1 | | |
| Paper Makers ... ... | 5 | Waistcoat Makers ... | 2 | | |
| Pew Openers ... ... | 2 | Washerwoman ... ... | 1 | | |
| Publican ... ... ... | 1 | Water Gilder ... ... | 1 | | |
| Servants ... ... ... | 296 | Weaver ... ... ... | 1 | | |
| Shoe Binders ... ... | 4 | Wig Maker ... ... | 1 | | |
| Shopkeepers ... ... | 17 | | | | |

Looking at the preceding table in the aggregate, clerks constitute the most numerous body of male patients admitted, whilst labouring under insanity. Next carpenters, labourers, and tailors; then turners, grocers, and schoolmasters; amongst the latter of whom there were twenty-seven instances. The circumstance seems rather remarkable, that so many teachers of youth as the number mentioned should have become insane, seeing schoolmasters are by no means a numerous fraternity. This is proved by the fact of there being only 1676 persons returned as so engaged by the census of 1851, and resident within the metropolis. Of course, it cannot hence be positively asserted that those engaged in teaching are more liable to become victims to mental disease than in some other occupations: as, for instance, medical practitioners, of whom twenty-two examples are reported to have been received into Bethlem Hospital, the total amount of physicians and surgeons being 3959 in London, or upwards double the number of schoolmasters. Again, thirty-four turners were admitted: and as this class is even less numerous than the latter, or only 1317 throughout the metropolitan districts, it seems not overstraining the argument to assume that individuals dedicated to this kind of employment become oftener insane than various other parties occupied in a different manner. For example, only thirty-five tailors are stated to have been received into the insane wards of Bethlem, notwithstanding that body of workmen is very numerous; there being not less than 20,257 in London, or more than fifteen times the number of turners; nevertheless, the total cases were almost identical.

Much the same kind of reasoning may also apply to servants, of which thirty-two cases of insanity are recorded; and as this class comprises 21,507 individuals, if those at inns are included, it becomes a circumstance worth noting that so few lunatics were comprised in this division. At all events, notwithstanding such inferences may appear rather more speculative than yet proved, the previous table certainly demonstrates, mental disease oftener supervenes amongst certain classes of workmen, compared with others, whose occupations are of a different description. In respect of turners, although a very limited body of artisans, it is somewhat singular, the cases registered were so numerous; and the above fact would almost warrant the conclusion that their particular kind of occupation apparently exerts an influence in producing these attacks. But whether through the rapid rotatory motion of the machinery used, and so exciting the brain, from the uniform attention required on the workman's part, or by the monotonous, but constantly changing aspect of the articles they make, deserves further attention and much additional experience before speaking upon the subject with confidence.

Amongst the female patients, the most numerous division of particular occupations is that of servants, of whom 296 cases attacked by insanity are so specified. Dress-makers, which includes milliners and needlewomen, also constitute a very numerous class; 212 examples being thus designated. Needlewomen and young girls employed in the sedentary occupation of sewing, and who are often very inadequately remunerated, appear much to be commiserated. That mental disease is by no means an uncommon occurrence amongst this unfortunate section of the female community, would seem undeniably demonstrated by the circumstance now mentioned. Another class of single women seems, however, even more predisposed to and afflicted by mental disease, namely, governesses: of whom sixty-two instances are recorded in the table; making nearly one in every thirty-three female lunatics admitted. Like schoolmasters, governesses are not a numerous body, speaking comparatively; and therefore the coincidence appears more singular, that both these classes, who are each engaged in training the rising generation, and also imparting knowledge to young minds, should respectively furnish so large a proportion of inmates to Bethlem Hospital; but why this remarkably similar result should happen is difficult of explanation. The anomalous condition in which governesses are generally placed, being neither ranked with domestic servants, nor usually allowed to associate with the masters or mistresses of the family where they reside, and are seldom permitted to mix in the ordinary society visiting the same domicile, whilst their own attention and time is constantly occupied in the harassing duties of teaching frequently wayward young girls, or noisy children, should be noted. Further, governesses being commonly women of talent and accomplishments, often reared in a different sphere to the one they then occupy, are obliged, in many instances, through family misfortunes, or poverty seldom occasioned by their own conduct, in order to earn a precarious livelihood, to accept appointments inferior in many respects even to lady's-maids or housekeepers. The latter are really servants, occupying their proper sphere, who generally never had enjoyed better treatment or experienced more prosperity, and are besides very rarely possessed of accomplishments or received a good education, hence, have their feelings seldom wounded from associating with individuals inferior to themselves; whereas, this too often happens in the case of parties under consideration. I scarcely know any class of society more deserving of sympathy than governesses, whether their anomalous social position be considered, or the frequency with which mental disease appears to supervene. Many afflicting cases of this description have been observed amongst the insane patients treated at Bethlem Hospital; and as similar examples are by no means rare in other institutions for lunatics, I have consequently been induced to enlarge upon such topics, in order to bring the question now discussed before the profession, and thus arrive at more correct deductions than the data here collected might yet seem to warrant.

Before bringing the present communication to a close, I would finally remark, that another class of females amongst whom insanity seems to have prevailed to some extent, considering their limited number as a distinct body, deserves a passing notice, namely, lodging-house-keepers. Of this body twenty-five instances are enumerated; and seeing the total lodging-house-keepers, male and female included, comprise only 553 individuals, throughout the metropolis, such a fact at least shows the ratio to be considerable. If, then, so many insane patients of that description come under treatment, the inference seems neither overstrained nor devoid of foundation, that females embraced by this particular division are oftener affected with mental disease than those enumerated within other categories; as, for example, midwives and washerwomen, only one instance of persons employed in each of these occupations being included in the previous table, although both these classes of women comprise rather a numerous body of individuals, throughout London and its immediate vicinity.

